# Carborane-Based ABCG2-Inhibitors Sensitize ABC-(Over)Expressing Cancer Cell Lines for Doxorubicin and Cisplatin

**DOI:** 10.3390/ph16111582

**Published:** 2023-11-09

**Authors:** Svetlana Paskas, Philipp Stockmann, Sanja Mijatović, Lydia Kuhnert, Walther Honscha, Evamarie Hey-Hawkins, Danijela Maksimović-Ivanić

**Affiliations:** 1 Department of Immunology, Institute for Biological Research “Siniša Stanković”, National Institute of Republic of Serbia, Belgrade University, 11060 Belgrade, Serbia; cecilijavo@googlemail.com (S.P.); sanjamama@ibiss.bg.ac.rs (S.M.); 2 Institute of Inorganic Chemistry, Faculty of Chemistry and Mineralogy, Universität Leipzig, Johannisallee 29, 04103 Leipzig, Germany; 3 Institute of Pharmacology, Pharmacy and Toxicology, Faculty of Veterinary Medicine, Universität Leipzig, An den Tierkliniken 15, 04103 Leipzig, Germany; lydia.kuhnert@vetmed.uni-leipzig.de (L.K.); honscha@vetmed.uni-leipzig.de (W.H.)

**Keywords:** breast cancer resistance protein, multidrug resistance, ABCG2, carborane

## Abstract

The ABCG2 transporter protein, as part of several known mechanisms involved in multidrug resistance, has the ability to transport a broad spectrum of substrates out of the cell and is, therefore, considered as a potential target to improve cancer therapies or as an approach to combat drug resistance in cancer. We have previously reported carborane-functionalized quinazoline derivatives as potent inhibitors of human ABCG2 which effectively reversed breast cancer resistance protein (BCRP)-mediated mitoxantrone resistance. In this work, we present the evaluation of our most promising carboranyl BCRP inhibitors regarding their toxicity towards ABCG2-expressing cancer cell lines (MCF-7, doxorubicin-resistant MCF-7 or MCF-7 Doxo, HT29, and SW480) and, consequently, with the co-administration of an inhibitor and therapeutic agent, their ability to increase the efficacy of therapeutics with the successful inhibition of ABCG2. The results obtained revealed synergistic effects of several inhibitors in combination with doxorubicin or cisplatin. Compounds **DMQCa**, **DMQCc**, and **DMQCd** showed a decrease in IC_50_ value in ABCB1- and ABCG2-expressing SW480 cells, suggesting a possible targeting of both transporters. In an HT29 cell line, with the highest expression of ABCG2 among the tested cell lines, using co-treatment of doxorubicin and **DMQCd**, the effective inhibitory concentration of the antineoplastic agent could be reduced by half. Interestingly, co-treatment of compound **QCe** with cisplatin, which is not an ABCG2 substrate, showed synergistic effects in MCF-7 Doxo and HT29 cells (IC_50_ values halved or reduced by 20%, respectively). However, a literature-known upregulation of cisplatin-effluxing ABC transporters and their effective inhibition by the carborane derivatives emerges as a possible reason.

## 1. Introduction

The term cancer is used for a large group of disorders characterized by the establishment of abnormal cell phenotypes leading to their uncontrolled growth and ability to infiltrate surrounding tissues and compromise their function. Many internal disorders together with external factors can result in genetic/epigenetic changes responsible for malignant alteration [1]. The comprehensive understanding of cancer as a disease and all factors that contribute to its progression resulted in the drafting of novel strategies in treatments, as well as the design of numerous small molecule therapeutics, from a non-selective to a targeted mode of action. Despite notable achievements in early detection and advanced treatment protocols, statistical data showcase that survival of patients with metastatic cancer is still low [2]. Multidrug resistance (MDR) is a condition in which cells become unresponsive to anticancer drugs, resulting in the failure of cancer therapy [3]. Various mechanisms contribute to chemotherapy resistance, such as genetic mutations [4,5], dysregulation of cell survival and death signaling pathways [6,7,8], increased drug efflux due to the overexpression of drug transporters [9], abnormality of cell repair systems [10], cancer stem cells enrichment [11,12,13], and epigenetic alterations [14].

MDR is commonly defined by the overexpression of ATP-binding cassette (ABC) transporters [15]. The ABC transporter superfamily consists of seven subfamilies from ABC A to ABC G [16], with the proteins being engaged in the absorption and secretion of endo- and exogenous substances [17]. Among the proteins known to induce MDR, the breast cancer resistance protein (BCRP) belongs to the ABC subfamily G, isoform 2, referred to as ABCG2. In healthy tissue, BCRP holds different roles such as protecting the fetus from endo- and exotoxins, protecting the fetal and postnatal brain from harmful compounds in the blood–brain barrier [18], or regulating the homeostasis of nutrients and their absorption in the gastrointestinal tract [19]. In cancer tissue, ABCG2 is responsible for the elimination of a variety of cytotoxic agents out of the cell, and the upregulated expression of this protein is associated with poor or failing response to chemotherapy [20,21,22,23]. ABCG2 transports a structurally diverse array of chemotherapeutic drugs, such as tyrosine kinase inhibitors [24], flavopiridol, camptothecins (like topotecan or irinotecan) [25], mitoxantrone, and anthracyclines (doxorubicin, daunorubicin) [26]. Considering the diversity of ABCG2 substrates, the list of inhibitors expands steadily. One of the most frequently used inhibitors is Ko143, an analog of fumitremorgin C, isolated from *Aspergillus fumigates* [27]. Further inhibitors are known, like elacridar and tariquidar, which are strong inhibitors of BCRP, but are not selective and also inhibit other ABC transporters [28]. However, none of the inhibitors targeting transporter proteins has been successfully evaluated in a clinical trial [29].

**Figure 1:**
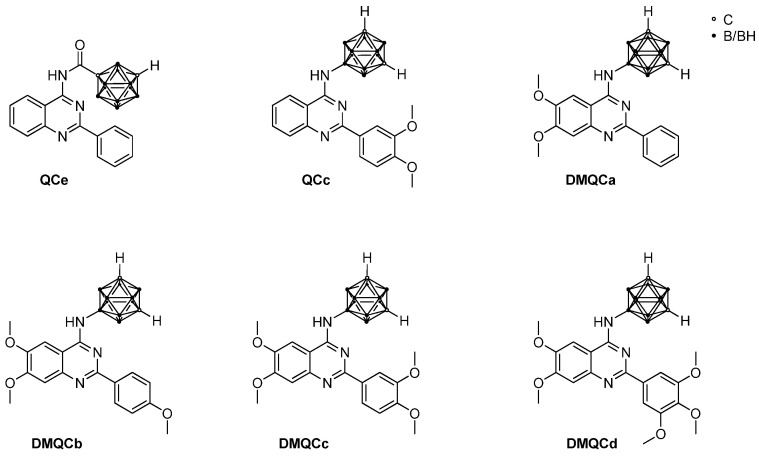
Molecular structures of *meta*-carboranyl quinazoline-based ABCG2 protein inhibitors [32].

There are three major strategies for handling ABC transporter-induced resistance: (1) pharmacological inhibition of ABCG2 activity, (2) inhibition of *ABCG2* expression, and (3) circumventing the ABCG2-mediated resistance by using agents that are poor substrates [29]. In our previous work [30,31,32] we have followed the strategy of synthesizing novel compounds able to inhibit the human ABCG2 protein. With the incorporation of a *meta*-carborane (*closo*-dicarbadodecaborane, C_2_B_10_H_12_) moiety as a pharmacophore into a (poly(methoxylated)) 2-phenylquinazolin-4-amine scaffold (Figure 1), potent, non-toxic inhibitors of BCRP were obtained; furthermore, a strong reversion of ABCG2-mediated mitoxantrone resistance in MDCKII-hABCG2 cells was achieved [32,33].

Carboranes, three-dimensional clusters, have become increasingly appealing in recent decades due to their unique and advantageous properties. By virtue of their high hydrophobicity, carboranes are attractive as pharmacophores for increasing membrane permeability [34]. Moreover, their inorganic nature provides an advantage of enhanced metabolic stability compared to organic analogues [35]. In particular, the use of carboranes as phenyl mimetics was previously shown to be beneficial for ABCG2 inhibition [30]. The *meta* isomer (*closo*-1,7-dicarbadodecaborane) is more stable than the *ortho* isomer (*closo*-1,2-dicarbadodecaborane) and was therefore chosen. Furthermore, it is far less expensive than the *para*-carborane (1,12-isomer), which is the most stable of the three isomers. Therefore, this work aims to elevate our previous findings by employing the strong carboranyl quinazoline inhibitors that were shown to reverse MDR in human *ABCG2*-overexpressing Madin-Darby canine kidney cells (MDCKII-hABCG2) [32,33], in co-administration with the BCRP substrate and chemotherapeutic agent doxorubicin on *ABCG2*-overexpressing cancer cell lines.

## 2. Results

Prior to the elucidation of the compounds’ influence on the cell viability, we have determined the levels of *ABCB1* and *ABCG2* expression in different cancer cell lines (Supplementary Materials Figure S1). The expression analysis was performed on the human melanoma cell line A375, three human colon carcinoma cell lines HT29, SW480, and SW620, as well as on two breast cancer cell lines MCF-7 and doxorubicin-resistant MCF-7 (MCF-7 Doxo). For further biological investigations, we have used cancer cell lines with different amounts of *ABCB1* and *ABCG2* expression levels. Therefore, HT29 (high expression of *ABCG2* compared to *ABCB1*), SW480 (approximately the same level of expression of both transporters), and MCF-7 and MCF-7 Doxo (increased *ABCG2* expression when resistance to doxorubicin is induced) cell lines were selected, while the cell lines A375 and SW620 were disregarded due to significantly higher levels of *ABCB1* compared to *ABCG2*.

**Figure 2:**
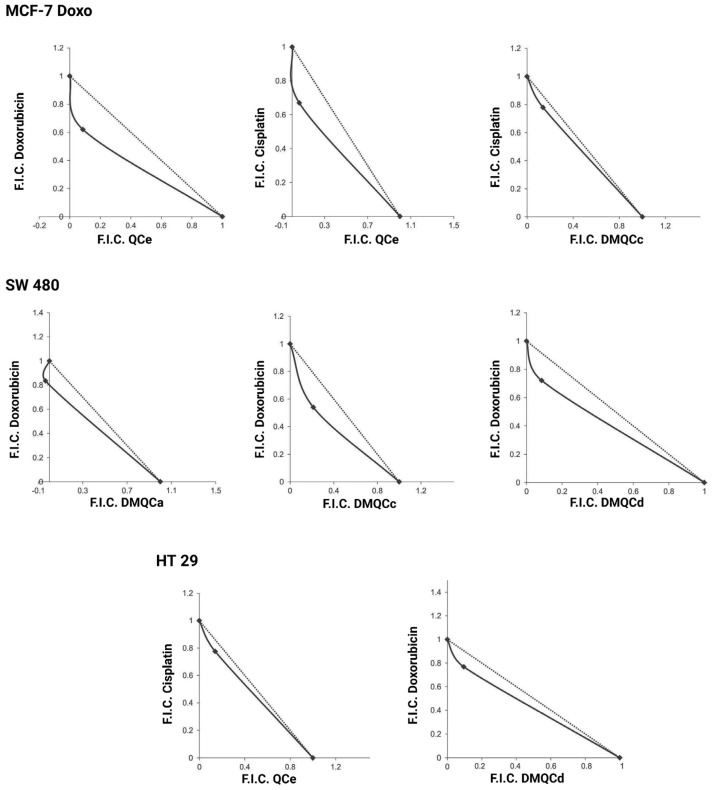
Isobologram analysis of combined treatment with synergistic interactions of ABCG2 inhibitors with doxorubicin and cisplatin in MCF-7 Doxo, SW480, and HT29 cell lines. One representative experiment out of two is presented. F.I.C. = fractional inhibitory concentration.

The inhibitors (Figure 1) were screened on the selected cell lines using the MTT (3-(4,5-dimethylthiazol-2-yl)-2,5-diphenyltetrazolium bromide) and CV (crystal violet) assays. Compounds **QCc**, **DMQCa**, **DMQCb**, **DMQCc**, and **DMQCd** exhibited no significant effect on the cell viability when applied in concentrations up to 50 µM (Figure S2, Supplementary Materials), while compound **QCe** showed high potency in MCF-7 and MCF-7 Doxo cell lines, with IC_50_ values of 30.7 ± 3.85 µM (MTT) and 24.85 ± 8.69 µM (CV) in MCF-7 cells, and 38.15 ± 2.75 µM (MTT) and 18.2 ± 4.52 µM (CV) in the doxorubicin-resistant cell line MCF-7 Doxo. The deviation of MTT and CV assay values suggests that compound **QCe** impedes mitochondrial respiration. As MTT and CV assays confirmed a cytotoxic effect of **QCe** on cancer cells, we lowered the applied concentration to reduce the toxic effect (viability > 80%) and thus observe solely the effect of the inhibition of the ABC transporter. As shown in the cytotoxicity experiments, the unsubstituted amide derivative **QCe** seems to be the most toxic compound in ABCG2-expressing cell lines, HT29, MCF-7, and MCF-7 Doxo (Figure S2), in comparison to **QCc** and polymethoxylated derivatives **DMQCc** and **DMQCd**. All investigated compounds exhibit an ABCG2 inhibition proven in MDCKII cells with a stable expression of human *ABCG2* [32,33]. It may be assumed that a lability of the amide functionality as well as divergent metabolic modification on an unsubstituted aromatic ring system (hydroxylation, etc.) over time on **QCe** may cause deviant toxicity, despite the structural similarity to the other tested compounds. Therapeutic treatment with ABCG2 inhibitors primarily aims to increase the sensitivity of cancer cells to cytostatic drugs or to reverse transporter-mediated drug resistance. Doxorubicin, a topoisomerase inhibitor, as a therapeutic agent was chosen to further establish a doxorubicin-resistant cell line. In addition, cisplatin, a DNA-binding agent widely used in the therapy of solid tumors without being a substrate of ABCG2, was used as a second cytostatic drug. Doxorubicin and cisplatin were thus used in mono- and combinational therapy. We co-administered the cytostatic drugs with ABCG2 inhibitors **QCc**, **QCe**, **DMQCa**, **DMQCb**, **DMQCc**, and **DMQCd** and used the isobologram analysis to visualize the response of the two-drug treatment (Figure 2). The summary of the drug–drug interactions is given in Table 1.

Compound **QCe** showed synergistic effects with doxorubicin and cisplatin in MCF-7 Doxo and cisplatin in HT29 cells. Compounds **DMQCa**, **DMQCc**, and **DMQCd** synergized with doxorubicin in the SW480 cell line. In addition, compound **DMQCc** exhibited synergism with cisplatin in MCF-7 Doxo cells. It is evident that the levels of synergy are cell line-specific and dependent on the drug combination. The calculated IC_50_ values of the individually administered therapeutics and in combination with the ABCG2 inhibitors are given in Table 2. In all synergistic combinations, we detected a decrease in the effective inhibitory concentration of the chemotherapeutic. Strikingly, a reduction of 50% was observed with the cisplatin–**QCe** co-treatment in MCF-7 Doxo, as well as the doxorubicin–**DMQCd** combination in SW480 and HT29 cell lines.

**Figure 3:**
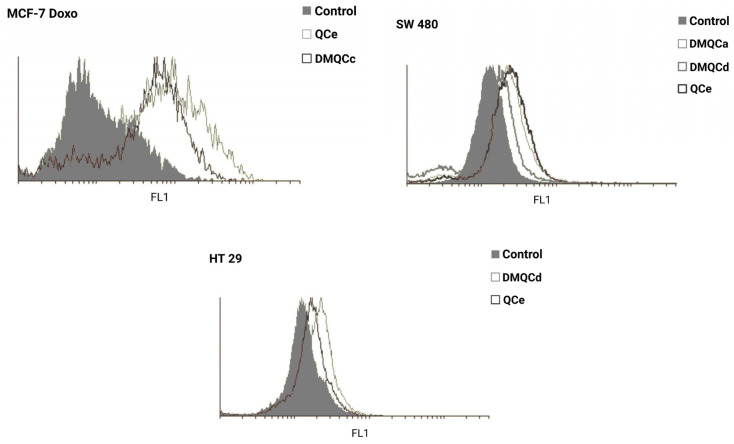
Functional analysis of the ABCG2 inhibitors using flow cytometry with JC-1 staining. The cells were treated with ABCG2 inhibitors for 48 h, trypsinized, and incubated with 2 µM JC-1 for 20 min at 37 °C. After incubation, the cells were washed and resuspended in cold PBS. One representative experiment out of three is shown.

Finally, ABCG2 inhibitors used in two-drug treatments were assessed for their ability to inhibit ABCG2 depending on the applied concentrations. Compounds **QCe**, **DMQCa**, **DMQCc**, and **DMQCd** were tested in cell lines in which a synergistic effect was observed. After 48 h incubation with the inhibitors, the cells were stained with ABCG2 marker dye JC-1 (tetraethylbenzimidazolylcarbocyanine iodide) and analyzed with flow cytometry (Figure 3). In particular, increased fluorescence in the treated samples, in comparison to the control, indicated dye retention and thus, the inhibition of ABCG2. Accordingly, we can conclude that the lower IC_50_ value of the chemotherapeutic agent in the combined treatment is a result of an effective ABCG2 inhibition.

Considering that the synergisms obtained are not consistent and comparable with results and trends of previous studies [32,33], docking studies on the cryo-electron microscopy (EM) structure of ABCG2 (pdb ID 5NJ3) [36] were performed to investigate possible differences between the binding behaviors and interactions of protein and substrate. Recently, we demonstrated a possible competitive inhibition mechanism between the carboranyl quinazoline BCRP inhibitors and the therapeutic drug mitoxantrone [32]. Accordingly, ‘blind’ dockings were conducted with a comparison of the inhibitors tested here and the ABCG2 substrate doxorubicin. The putative binding poses obtained exhibited an affinity of doxorubicin in the lateral binding pocket S2 of the inner cavity (see Figure S3, Supplementary Materials). The inhibitors, as previously reported, showed strong binding in the central slit-like binding pocket S1. Further details and representations are given in the Supplementary Materials.

## 3. Discussion

Chemotherapy is still one of the major components of cancer treatment. Nowadays, the combination of drugs gives rise to new approaches towards targeting more specific therapies, and with that, for example, opens the possibility to overcome the resistance to given treatments. The main goal when designing a combined treatment is to achieve synergy by increasing the efficacy and reducing the toxicity of individual drugs. In this work, we evaluated the influence of co-administration of ABCG2 inhibitors and antineoplastic agents in ABCG2-expressing cell lines. Successful inhibition of the transporter yields an intracellular increase in therapeutic drug concentration and, thus, increased efficacy or reversal of efflux protein-induced resistance to therapeutic drugs. Our previous data showed that the introduction of a carborane moiety into a polymethoxylated 2-phenylquinazolin-4-amine scaffold generated strong BCRP inhibitors that potently reversed ABCG2-mediated mitoxantrone resistance [32,33,34,37,38,39]. In different studies, we similarly observed an enhanced ABCG2 inhibition using carboranes as phenyl mimetics in baicalein derivatives [30]. As an in vitro model of chemoresistance, we used colon and breast carcinoma cell lines, and the MCF-7 Doxo cell line, in which the *ABCG2* expression was proven to be upregulated, as further reported by several independent studies [40,41,42].

Cell viability assays revealed a significant difference between **QCe** and the other examined carborane derivatives. While **QCe** affected the viability of cancer cells in lower micromolar concentrations, no to low toxicity in the tested ranges was observed for compounds **QCc**, **DMQCa**, **DMQCb**, **DMQCc**, and **DMQCd**. These results are in agreement with recently reported findings on MDCKII cells [33] and similarly suggest a reduced influence on cell viability in cancer cells of *N*-carboranyl quinazolines and a polymethoxylated substitution pattern compared to the unsubstituted amide derivative **QCe**, validating the general stability and decreased toxicity of carborane derivatives compared to their organic analogues [33,43,44]. In general, the effect of chemoresistance reversal was observed in several studies and different cancer types. For example, Yin et al. [44] demonstrated that both pharmacological and siRNA inhibition of *ABCG2* leads to the reversal of the chemoresistance of liver cancer stem cells. Shivhare and Das [43] reported the reduction of the inhibitory concentration of doxorubicin and tamoxifen in an in vitro breast cancer study by combining the chemotherapeutics with a pan-ABC transporter inhibitor. Furthermore, in a study on small cell lung cancer, treatment with the ABC transporter inhibitors elacridar and tariquidar restored the cells’ sensitivity to topoisomerase inhibitors [45].

As our previous studies ascertained, the inhibitors **QCe**, **DMQCc**, and **DMQCd** successfully increased the sensitivity of MDCKII cells, overexpressing the human *ABCG2*, to mitoxantrone [32]. Herein, we shifted the experimental setting to human cancer cell lines and combined treatment of ABCG2 inhibitors and cytostatic drugs, displaying three synergistic combinations in MCF-7 Doxo, three in SW480, and two in HT29 cell lines. In consequence, we successfully lowered the effective concentration of the applied chemotherapeutics via combination with the novel hybrid inorganic–organic carboranyl quinazoline-based ABCG2 inhibitors **QCe**, **DMQCc**, and **DMQCd**. However, the results obtained are not consistent with the co-administration of the inhibitors with mitoxantrone. Differences in the transporter proteins may occur since a multitude of mutations of human ABCG2 are known [46]. In consideration of the molecular docking results, mitoxantrone and doxorubicin appear to bind in different cavities within the inner binding pocket of the human ABCG2 transporter. Mitoxantrone exhibits a similar binding mode as detected for quinazoline derivatives, with π-π stacking between the opposing Phe439 amino acid residues and a hydrogen bond towards Asn436 [47]. Therefore, in our previous results, a competitive inhibition of the mitoxantrone by carborane-based derivatives was assumed [32,33]. An inhibitor, located between Phe439-Phe439′ in the inward-facing state of ABCG2 is described as the most common inhibition mechanism preventing the conformational change to the outward-facing state. As speculated for lapatinib [48], a potential inhibition of the clamp by **DMQCc** and **DMQCd** through π-π stacking can be suggested [32]. In contrast, doxorubicin binds to a lateral pocket within the inner binding pocket. Thus, a conformational change induced by the inhibitor is needed to prevent the doxorubicin efflux and reverse chemotherapy resistance. The binding of an inhibitor to Asn436 is known to stabilized the inward-facing state of ABCG2 which inhibits the transport of hydrophilic ABCG2 substrates [48]. Even if the docking of our carborane-based compounds predicted a hydrogen bond towards Asn436, it seems not to influence the conformational change of the protein. Therefore, no synergistic effect with doxorubicin was detectable. We thus further hypothesized, by means of in silico studies and comparison of the putative docking poses of the therapeutic drug and the inhibitors, a possible non-competitive mechanism. Based on our previous results, competitive inhibition with mitoxantrone was revealed [32]. This might indicate a substrate-specific resistance reversal; however, further investigation is required.

Surprisingly, strong synergy was observed with co-treatment of cisplatin with **QCe** and **DMQCc** in MCF-7 Doxo cells. Cisplatin is known to be no substrate for ABCG2 and is often used as a positive control in the literature, yet it is a substrate for ABCC2, ABCC5, and ABCC6 [49]. Chen et al. [50] further reported the significant upregulation of *ABCC2* and *ABCC5* in doxorubicin-resistant MCF-7 cells, implying, based on the observed synergy, a non-ABCG2-specific inhibition. Therefore, it is suspected that compounds **QCe** and **DMQCc** are able to inhibit cisplatin-extruding ABC transporters, and thus increase the sensitivity to cisplatin. Further biological studies on corresponding targets such as ABCC transporters [50] are needed; however, they exceed the scope of this work. More importantly, these data strongly suggest that a multi-target chemotherapy regiment consisting of doxorubicin and cisplatin will unlikely have a positive outcome, as doxorubicin treatment induces the upregulation of transporters for both chemotherapeutics. There are several clinical studies in which therapy failure can be explained by these findings [51,52,53]. In general, major problems with the introduction of MDR inhibitors in cancer treatment as chemo-sensitizing agents are connected with the high toxicity of nonselective molecules and the lack of efficacy of highly selective forms as a consequence of substrate overlapping. The data presented here appear useful for the modification of chemotherapeutic regimens or schedules. Based on our in vitro results, in particular the ability of non-toxic carborane-based ABCG2 inhibitors to affect ABC transporter-associated doxorubicin and cisplatin efflux, thus sensitizing the examined cancer cells for the applied therapeutics, future in vivo studies are of interest.

## 4. Materials and Methods

### 4.1. Reagents and Cells

Fetal calf serum (FCS), RPMI-1640 medium, phosphate buffer saline (PBS), and dimethyl sulfoxide (DMSO) were from Merck (Darmstadt, Germany). A375, HT29, SW480, SW620, and MCF-7 cell lines were purchased from American Type Culture Collection (Rockville, MD, USA). Cells were routinely maintained in 4-(2-hydroxyethyl)-1-piperazineethanesulfonic acid (HEPES)-buffered RPMI-1640 medium supplemented with 10% FCS with 2 mM L-glutamine, 0.01% sodium pyruvate, 100 U/mL penicillin, and 100 μg/mL streptomycin. All cell lines were cultured in a 5% CO_2_ and humid atmosphere.

### 4.2. Establishment of the Doxorubicin-Resistant MCF-7 (MCF-7 Doxo) Cell Line

The doxorubicin resistance was induced by persistent treatment of MCF-7 cells with doxorubicin, in concentrations rising from 10 nM to 100 nM, as described by Marinello et al. [54]. The cells were seeded in the T25 flask. When they were approximately 80% confluent, doxorubicin was added in a final concentration of 10 nM. The medium was changed every 2 to 3 days, adding fresh doxorubicin in a rising concentration. Non-treated MCF-7 cells were grown as a control in the same cell passage as Doxo-treated cells.

After 6–8 weeks, the IC_50_ values for control and Doxo-treated cells were measured. In every preparation, the IC_50_ of Doxo-treated cells was approximately 10 times higher than in the control.

### 4.3. Gene Expression Analysis

RNA isolation, cDNA synthesis, and qRT-PCR were carried out as described by Vesel et al. [55]. We have used the *ABCB1* and *ABCG2* gene-specific primers from the same publication.

RNA was isolated using TRI Reagent (Sigma-Aldrich, St. Louis, MO, USA). After quantification using Nanodrop, 1 µg of RNA was used for the RT reaction, together with random hexamer primers, RiboLock, and reverse transcriptase enzyme (all from Thermo Scientific, Waltham, MA, USA). qPCR was performed using SYBR Green chemistry (Thermo Scientific, Waltham, MA, USA).

### 4.4. Cell Viability

In brief, 4000 cells were seeded in 96-well plates in 100 µL volume, and treated with different concentrations of ABCG2 inhibitors for 48 h. For the crystal violet (CV) assay, the cells were fixed and incubated for 15 min at room temperature with 1% crystal violet (Mol, Belgrade, Serbia). The absorbance of dissolved dye was measured at 540 nm. Cell viability was calculated as a percentage of untreated wells. For the MTT (3-(4,5-dimethylthiazol-2-yl)-2,5-diphenyltetrazolium bromide) assay, the mitochondrial dehydrogenase activity was determined by the reduction of MTT to formazan. Cells were treated with 0.5 mg/mL MTT (Sigma, St. Louis, MO, USA) and incubated at 37 °C. When the color of the solution changed from yellow to brown, DMSO was added and the absorbance was measured at 540 nm. Cell viability was calculated as a percentage of control that was arbitrarily set to 100%.

### 4.5. Isobologram Analysis

A total of 4000 cells were seeded in 96-well plates in 100 µL volume, in an RPMI medium supplemented with 10% FCS with 2 mM L-glutamine, 0.01% sodium pyruvate, 100 U/mL penicillin, and 100 μg/mL streptomycin. The cells were treated with four different concentrations of ABCG2 inhibitors (0, 1.5, 3.12, and 6.25 µM) and doxorubicin (0, 0.25, 0.5, and 1 µM), or cisplatin (0, 7.5, 15, and 30 µM) for 48 h. The concentrations of cytostatic drugs were chosen based on our previous results [56]. Cells were treated with 0.5 mg/mL MTT (Sigma, St. Louis, MO, USA) and incubated at 37 °C. When the color of the solution changed from yellow to brown, DMSO was added and the absorbance was measured at 540 nm. Cell viability was calculated as a percentage of control that was set to 100%. The IC_50_ values of single and combined treatment were compared and the isobologram analysis was performed as described by Tallarida et al. [57]. The isobologram curves were constructed for the drug combinations where the synergistic effect was observed.

### 4.6. JC-1 Staining

Dye loading and flow cytometry was carried out as described by Wolosin et al. [58]. The cells were seeded in a 6-well plate (250,000 cells/well in 1 mL volume), and treated with ABCG2 inhibitors for 48 h, trypsinized, and incubated with 2 µM JC-1 for 20 min at 37 °C. After incubation, the cells were washed and resuspended in cold PBS (phosphate-buffered saline, pH 7.2). Analytical flow cytometry was performed on CyFlow Space (Partec, Münster, Germany).

### 4.7. Statistical Analysis

We used the Statistical Package for the Social Sciences (SPSS, IBM, Armonk, NY, USA) for data analysis. The Student’s *t*-test, Mann–Whitney test, and one-way ANOVA (Tukey’s test as post hoc) were employed to evaluate the significance between groups. Differences were considered significant when the *p* value was less than 0.05.

### 4.8. Molecular Docking

In order to assess the putative binding modes of the examined compounds (**QCc**, **QCe**, **DMQCa**, **DMQCb**, **DMQCc**, **DMQCd**, and doxorubicin), molecular modeling analysis was carried out after a recently published protocol [28].

## 5. Conclusions

The inhibition of drug secretion by MDR transporters is a powerful tool in the creation of novel therapeutic protocols. Here we reported the effect of carborane-containing therapeutics—ABCG2 inhibitors that were effective in low dosage, non-toxic to cancer cells, and performed in synergy when combined with doxorubicin and cisplatin. ABCG2-inhibiting compound **QCe** exhibited further synergistic effects with doxorubicin and cisplatin in ABCG2-expressing cancer cells. Furthermore, the synergistic effects of **DMQCa**, **DMQCc**, and **DMQCd** in combination with a chemotherapeutic, doxorubicin, for different cancer cells were shown. The data of this work yielded a selection of four promising candidates worthy of future investigation. Keeping in mind that an in vitro system is simplified at multiple levels is of pivotal interest to explore the reproducibility of effects observed in cell culture in animal models as a key step prior to clinical trials.

## Figures and Tables

**Table 1:** Overview of the synergistic (Syn) or antagonistic (Ant) interactions of the co-treatment of ABCG2 inhibitors **QCc**, **QCe**, **DMQCa**, **DMQCb**, **DMQCc**, and **DMQCd** with doxorubicin or cisplatin. Doxo = doxorubicin; CisPt = cisplatin.

Compound	MCF-7		MCF-7 Doxo		SW480		HT29	
	Doxo	CisPt	Doxo	CisPt	Doxo	CisPt	Doxo	CisPt
**QCc**	Ant	Ant	Ant	Ant	Ant	Ant	Ant	Ant
**QCe**	Ant	Ant	**Syn**	**Syn**	Ant	Ant	Ant	**Syn**
**DMQCa**	Ant	Ant	Ant	Ant	**Syn**	Ant	Ant	Ant
**DMQCb**	Ant	Ant	Ant	Ant	Ant	Ant	Ant	Ant
**DMQCc**	Ant	Ant	Ant	**Syn**	**Syn**	Ant	Ant	Ant
**DMQCd**	Ant	Ant	Ant	Ant	**Syn**	Ant	**Syn**	Ant

**Table 2:** IC_50_ values of chemotherapeutics doxorubicin and cisplatin with and without co-administration of ABCG2 inhibitors.

Cell Line	Therapeutic	Inhibitor	IC_50_ (Therapeutic) [µM]	IC_50_ (Therapeutic + Inhibitor) [µM]
**MCF-7 Doxo**	Doxorubicin	**QCe**	1.15 ± 0.20	0.80 ± 0.23
	Cisplatin	**QCe**	25.10 ± 0.06	12.05 ± 1.30
	Cisplatin	**DMQCc**	26.65 ± 0.43	19.65 ± 0.03
**SW480**	Doxorubicin	**DMQCa**	1.25 ± 0.20	0.95 ± 0.14
	Doxorubicin	**DMQCc**	0.60 ± 0.00	0.45 ± 0.03
	Doxorubicin	**DMQCd**	0.90 ± 0.10	0.45 ± 0.08
**HT29**	Cisplatin	**QCe**	25.05 ± 2.05	19.50 ± 1.16
	Doxorubicin	**DMQCd**	1.00 ± 0.05	0.45 ± 0.08

## Data Availability

Data is contained in the paper.

## Supplementary Materials

The following supporting information can be downloaded at: https://www.mdpi.com/article/10.3390/ph16111582/s1. Biological data (expression of *ABCB1* and *ABCG2* in human cancer cell lines; influence of **QCc**, **QCe**, **DMQCa**, **DMQCb**, **DMQCc**, and **DMQCd** on the viability of human carcinoma cell lines) and computational data (binding poses of doxorubicin, **QCe**, **DMQCc**, and **DMQCd** in the ABCG2 cryo-EM structure). References [32,33,36] are cited in the Supplementary Materials.
