# Supplementary material for: Carborane-Based ABCG2-Inhibitors Sensitize ABC-(Over)Expressing Cancer Cell Lines for Doxorubicin and Cisplatin
Source: Pharmaceuticals (Basel). 2023 Nov 9;16(11):1582. doi: 10.3390/ph16111582 (PMC10674596; doi:10.3390/ph16111582)
Supplement: Supplementary file 1 [file pharmaceuticals-16-01582-s001.zip › pharmaceuticals-2647030-supplementary.pdf]

Supplementary Materials

Carborane-based ABCG2-inhibitors sensitize ABC-(over)expressing cancer cell lines for doxorubicin and cisplatin

Svetlana Paskas, Philipp Stockmann, Sanja Mijatović, Lydia Kuhnert, Walther Honscha, Evamarie Hey-Hawkins and Danijela Maksimović-Ivanić

This file includes

1. Biological Data
(Figures S1 and S2)

2. Computational Data
(Figure S3)

1. Biological Data

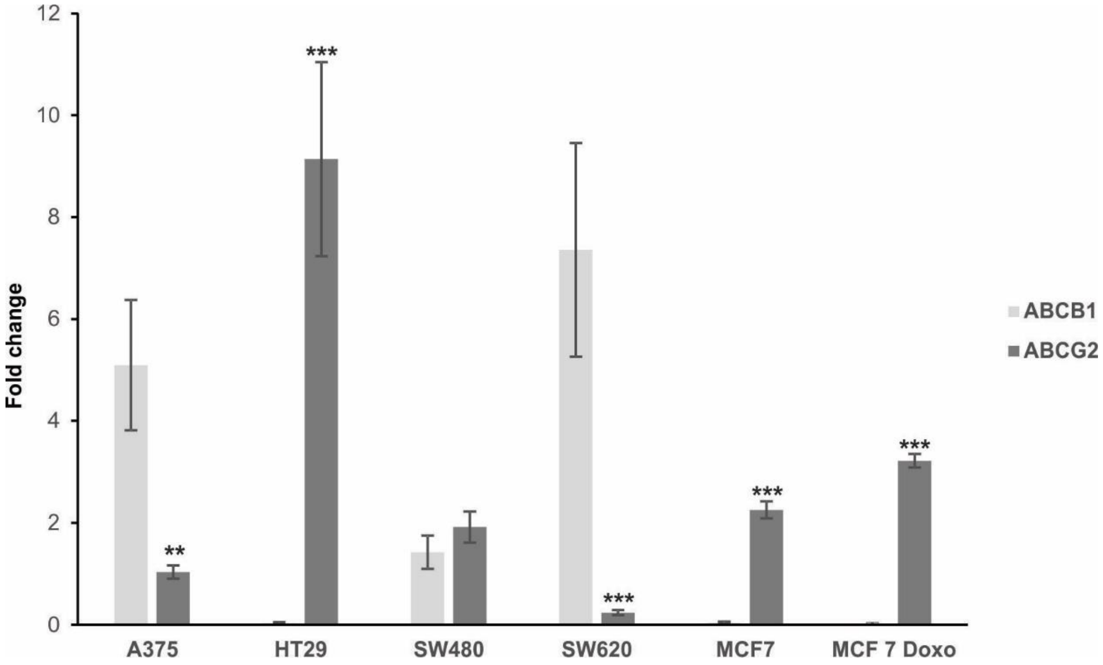

Figure S1: Expression of ABCB1 and ABCG2 in human cancer cell lines. The total mRNA was isolated from A375, HT29, SW480, SW620, MCF-7, and MCF-7 Doxo cell lines. The expression of ABCB1 and ABCG2 was firstly normalized to GAPDH and then to ABCG2 expression in A375 cell line. The results are presented as means of fold change± SEM from five independent experiments. * significant difference of ABCB1 mRNA expression in comparison to ABCG2 in each cell line, tested by one-way ANOVA using Tukey's test as post-hoc.

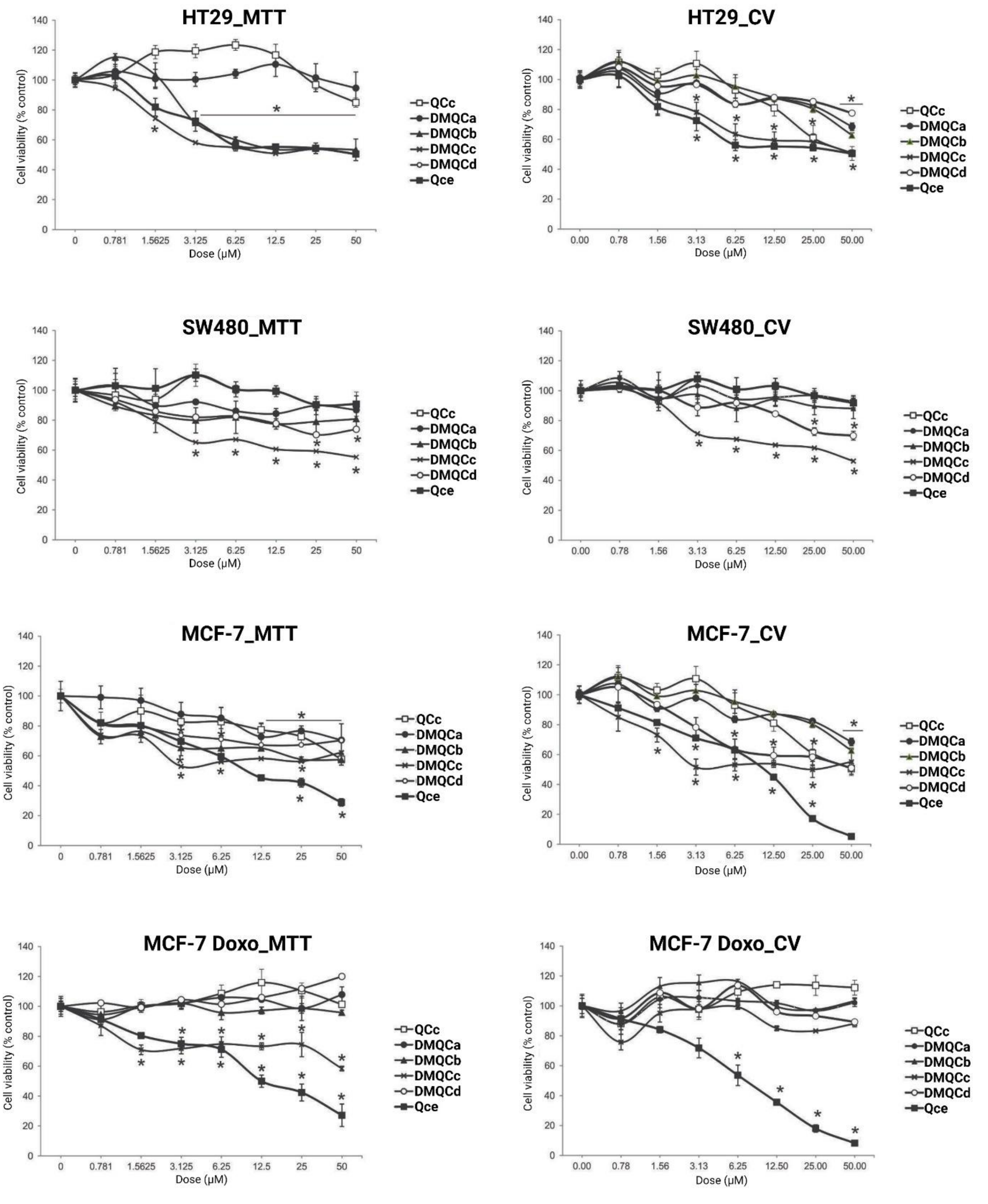

Figure S2: Human colon carcinoma (HT29, SW490) and human breast cancer (MCF-7 and MCF-7 Doxo) cell lines were treated with **QCc**, **QCe**, **DMQCa**, **DMQCb**, **DMQCc**, **DMQCd** in concentrations ranging from 0 to 50 μM for 72 h. Cell viability was determined by the MTT and CV assay. The data are presented as a percentage of control± SD from one representative out of three independent experiments. *p<0.05 refers to untreated cultures.

2. Computational Data

Docking simulations as well as structure and protein preparation were carried out after a recently published protocol [1].

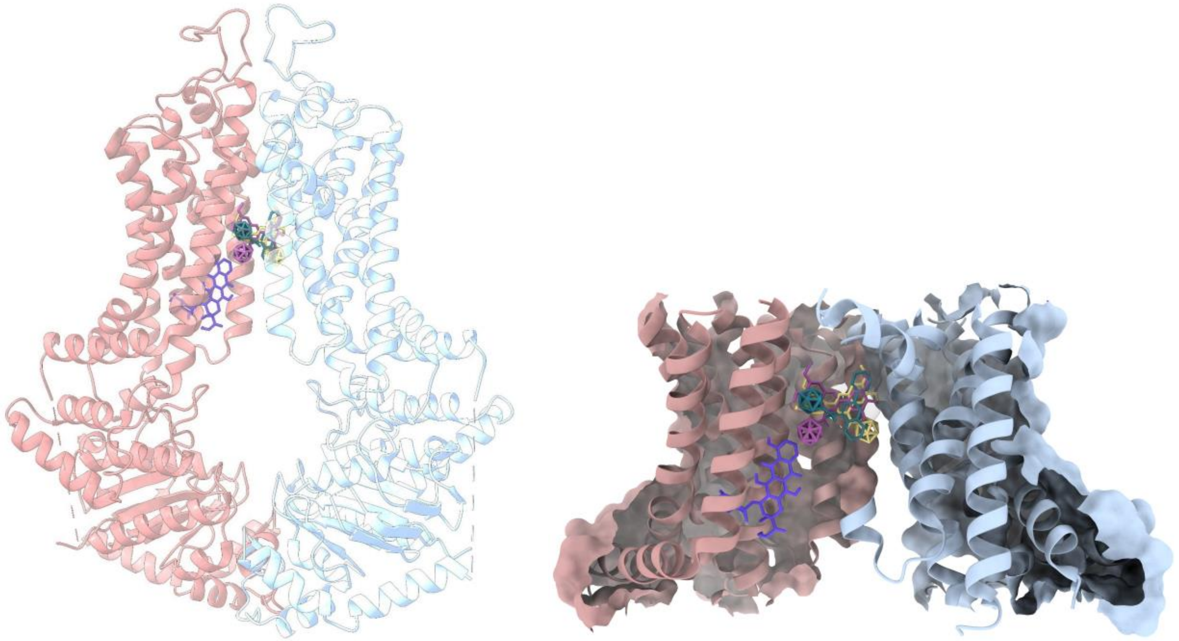

Figure S3. Left: Binding poses of doxorubicin (blue), **QCe** (green), **DMQCc** (yellow) and **DMQCd** (purple). Monomers colored rose and blue. Right: Top-ranked poses of doxorubicin (blue), **QCe** (green), **DMQCc** (yellow) and **DMQCd** (purple) docked into the ABCG2 cryo-EM structure (PDB code 5NJ3 [2]); compounds shown as stick model; hydrogen atoms omitted for clarity.

The calculated binding poses of the carboranyl derivatives **QCe**, **DMQCc** and **DMQCd** predominantly (>60% of the top 100 docking poses of **QCe** and >90% for **DMQCc** and **DMQCd**) occupy the inner binding pocket S1, exhibiting, however, deviations in their orientation within the binding pocket. As the carborane cluster of the *N*-carboranyl quinazolines protrudes from the inward-facing cavity, their unsubstituted amide analogue faces the steric moiety in an outward-facing manner. Yet, the bicyclic quinazoline structure of all the examined compounds is found in the gap between the two opposing phenylalanine moieties (Phe439) of the two monomers. Doxorubicin, on the other hand, occupies in 100% of the found binding modalities the lateral pocket (see Figure S3), evincing, however, no favored and prevalent binding modality within the cavity. The calculated binding free energy values are given in Table S1.

Table S1. Free binding energies towards human ABCG2 transporter in the rigid ABCG2 protein (PDB code 5NJ3). Values of **QCe**, **DMQCc** and **DMQCd** are taken from previous publications [1,3].

Compound	Free binding energy [kcal/mol]
**Doxorubicin**	–3.4
**QCe**[3]	–7.1
**DMQCc**[1]	–10.4
**DMQCd**[1]	–8.1

3. References

1. Stockmann, P.; Kuhnert, L.; Leinung, W.; Lakoma, C.; Scholz, B.; Paskas, S.; Mijatović, S.; Maksimović-Ivanić, D.; Honscha, W.; Hey-Hawkins, E. The More the Better-Investigation of Polymethoxylated N-Carboranyl Quinazolines as Novel Hybrid Breast Cancer Resistance Protein Inhibitors. *Pharmaceutics.* **2023**, *15*, 241. https://doi.org/10.3390/pharmaceutics15010241.
2. Taylor, N.M.I.; Manolaridis, I.; Jackson, S.M.; Kowal, J.; Stahlberg, H.; Locher, K.P. Structure of the human multidrug transporter ABCG2. *Nature* **2017**, *546*, 504–509. https://doi.org/10.1038/nature22345.
3. Stockmann, P.; Kuhnert, L.; Krajnović, T.; Mijatović, S.; Maksimović-Ivanić, D.; Honscha, W.; Hey-Hawkins, E. Carboranes as potent phenyl mimetics. A comparative study on the reversal of ABCG2-mediated drug resistance by carboranylquinazolines and their organic isosters. *ChemMedChem.*, 1–19 (submitted).
